# Reducing Visceral Leishmaniasis by Insecticide Impregnation of Bed-Nets, Bangladesh

**DOI:** 10.3201/eid1907.120932

**Published:** 2013-07

**Authors:** Dinesh Mondal, M. Mamun Huda, Mithun Kumar Karmoker, Debashis Ghosh, Greg Matlashewski, Shah Golam Nabi, Axel Kroeger

**Affiliations:** International Centre for Diarrhoeal Disease Research, Bangladesh, Dhaka, Bangladesh (D. Mondal, M.M. Huda, M.K. Karmoker, D. Ghosh);; World Health Organization, Geneva, Switzerland (G. Matlashewski, A. Kroeger);; Directorate General of Health Services of the Government of Bangladesh, Dhaka (S.G. Nabi);; Liverpool School of Tropical Medicine, Liverpool, UK (A. Kroeger)

**Keywords:** Bangladesh, visceral leishmaniasis, vector control, bed-net impregnation, vector-borne infections, insecticides, Leishmania spp., parasites, sandflies

## Abstract

The effect of insecticide-treated materials on reducing visceral leishmaniasis (VL) is disputable. In Bangladesh, we evaluated the effect of a community-based intervention with insecticide impregnation of existing bed-nets in reducing VL incidence. This intervention reduced VL by 66.5%. Widespread bed-net impregnation with slow-release insecticide may control VL in Bangladesh.

The governments of Bangladesh, India, and Nepal have committed to eliminate visceral leishmaniasis (VL) by 2015 (*1*). Reducing VL incidence by controlling sandflies, the vector of *Leishmania* spp. parasites, through integrated vector management is a key strategy of elimination programs (*2*). Community-based intervention with insecticide-treated materials, such as distribution of long-lasting insecticide–treated bed-nets or mass bed-net impregnation programs with slow-release insecticide tablets, could be possible vector-control components of integrated vector management if they are found effective in reducing VL incidence (*3*). We evaluated the effect of a community-based intervention with impregnation of existing bed-nets in reducing VL incidence in VL-endemic villages of subdistrict (upazila) Godagari, district Rajshahi, Bangladesh.

## The Study

**Figure 1:**
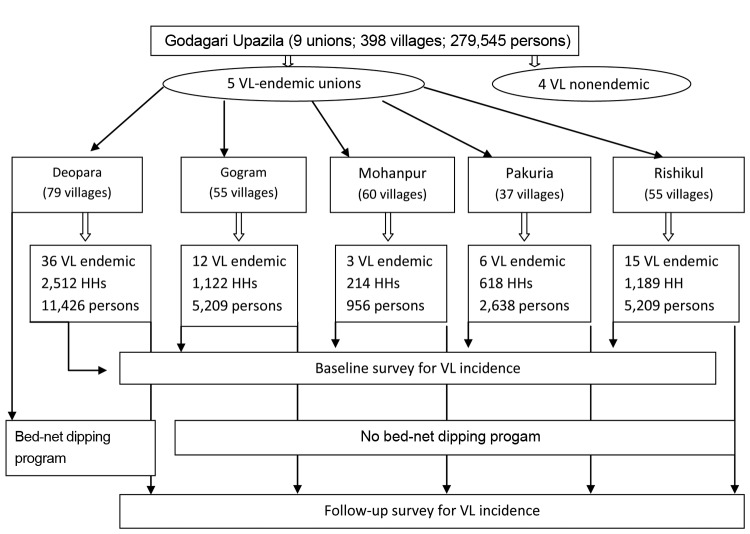
Design of study of reducing visceral leishmaniasis by insecticide impregnation of existing bed-nets, Bangladesh, 2006–2010. VL, visceral leishmaniasis; HH, households.

The study comprised all 72 VL-endemic villages in Godagrai, distributed in 5 unions (Deopara, 36; Rishikul, 15; Gogram, 12; Pakuria, 6; and Mohanpur, 3). The intervention area was 36 villages in Deopara union comprising 2,512 households (11,426 inhabitants), and the control area was the 36 villages from other 4 unions comprising 3,143 households (14,021 inhabitants) (Figure 1). The bed-net impregnation intervention program with KO Tab 1-2-3 (Bayer Environmental Science, Bayer [Ply] Ltd., reg. no. 1968/011192/07, 21 Isando, South Africa, CODE 05682036 C) was conducted during February–March 2008. All households from all 79 villages in Deopara union, including households in 36 VL-endemic villages, were invited to participate in bed-net dipping (Figure 1). Details about the surveys and intervention are given in the Technical Appendix. We measured VL incidence in the intervention and control areas before and after intervention during September 2006–March 2007 and December 2009–January 2010, respectively. Household screening for VL cases in the previous 12 months was performed by trained field research assistants. Past VL cases were confirmed through document analysis and checking of hospital registers. A new VL case was defined by using the definition for new VL case of the National Kala-azar Elimination Guideline (*4*). VL incidence was expressed by number of VL cases (newly found plus past VL cases) per 10,000 persons. The field research assistants also conducted an in-depth interview with each household head by using a structured questionnaire in every 11th household and in households where they found new and past VL cases to collect sociodemographic characteristics of the surveyed community and VL-related knowledge and practice. A total of 556 household heads (254 and 302, respectively, in the intervention and control areas) were interviewed. Sociodemographic and knowledge, attitude, and practice variables between 2 areas with p values <0.2 were extended to 5,655 households by using statistical tools, and the validity was checked by comparing the distribution of each variable before and after random extension (Technical Appendix Table). This helped us to investigate the eventual confounding effect of socioeconomic and knowledge, attitude, and practice variables on VL incidence reduction.

We evaluated the effect of the intervention on VL incidence in different ways. First, we compared reduction of VL incidence at the population level. Second, we compared reduction of VL-affected households in the 2 areas by a difference-in-difference method. Then, we examined the consistency of the effect of the intervention by measuring protection of the population from VL in the intervention area and protection of households from VL by the intervention through unadjusted and adjusted longitudinal logistic regression models. Data management and statistical analysis were conducted by using Epi Info version 3.2.2 (Centers for Disease Control and Prevention, Atlanta, GA, USA) and Stata 10.1 (Stata Corp, College Station, TX, USA), respectively. The International Centre for Diarrhoeal Disease Research, Bangladesh, and the Ethical Review Committees of the Special Program for Research and Training in Tropical Diseases/World Health Organization (WHO) approved the study. Informed written consent was obtained from each household head and from the persons with suspected VL for any study-related interventions.

The 2 areas differed regarding knowledge of the household head about VL symptoms, VL transmission, and household education (Technical Appendix). A total of 2,239 (89.1%) of the 2,512 household heads from the study area of Deopara participated in the bed-net dipping. The use of impregnated bed-nets was also very high (99.8%), as found by random nightly observation in a subsample of households in the intervention area.

**Figure 2:**
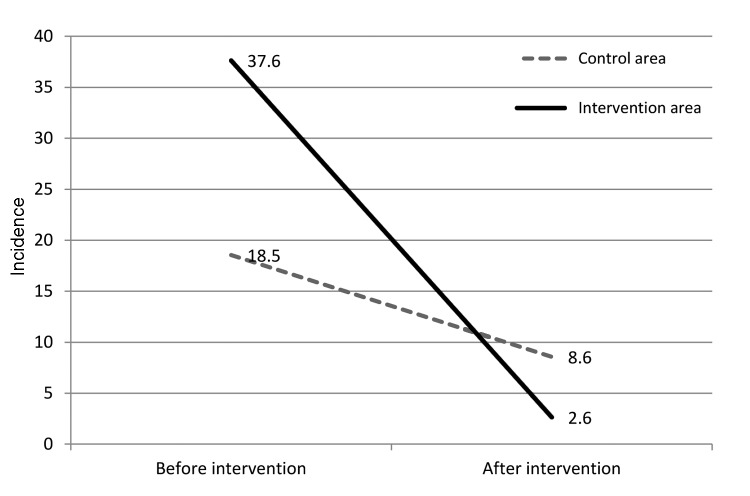
Visceral leishmaniasis incidence (cases per 10,000 persons) in intervention and control areas before and after intervention, Bangladesh, 2006–2010.

Before intervention, 69 VL cases were found, resulting in a VL incidence of 27 per 10,000 persons in the study area. VL incidence in the intervention area, 37.6 cases per 10,000 persons (43/11,426), was significantly higher than in the control area (18.5/10,000) (26/14,021; p = 0.0036). In intervention and control areas, 3 and 4 households, respectively, had multiple persons with VL. After intervention, VL incidence in intervention and control areas was 2.6 (3/11,426) and 8.6 (12/14,021) cases per 10,000 persons, respectively. During follow up, annual VL incidence declined in both areas, but the reduction was significantly greater in the intervention area (decrease of 35 cases/10,000 persons) than in the control area (decrease of 9.99/10,000; p = 0.001) (Table 1; Figure 2). The effect of community-level intervention, measured by difference-in-difference method, was 66.5% (Table 1). Using odds ratios in the longitudinal logistic regression model, we found that 85.8% (95% CI 44.0%–96.5%; p = 0.005) of the population in the intervention area was protected from VL by the intervention.

**Table 1:** VL incidence and affected households before and after bed-net impregnation program, Bangladesh, 2006–2010*

Group	Bed-net impregnation		Rate changes (p value)	% Reduction† compared with control (p value)‡
	Before, no. (%) affected	After, no. (%) affected		
HH§				
Intervention, n = 2,512	40 (15.92)	3 (1.19)	–14.73 (<0.0001)	–70.52% (0.0007)
Control, n = 3,143	21 (6.68)	10 (3.18)	–3.50 (0.0476)	
Total, n = 5,655	61 (10.79)	13 (2.30)	–8.49 (<0.0001)	
Population¶				
Intervention, n = 11,426	43 (37.63)	3 (2.63)	–35.01(<0.0001)	–66.49% (0.001)
Control, n = 14,021	26 (18.54)	12 (8.56)	–9.99 (0.023)	
Total, n = 25,447	69 (27.12)	15 (5.89)	–21.22 (<0.0001)	

*VL, visceral leishmaniasis; HH, households. †Effect of intervention: (B/A) – (D/C) Where A = baseline value for VL-affected HH per 1,000 HH/VL incidence per 10,000 persons in the intervention area; B = post-intervention value for VL-affected HH per 1,000 HH/VL incidence per 10,000 persons in the intervention area; C = baseline value for VL-affected HH per 1,000 HH/VL incidence per 10,000 persons in the control area; D = post-intervention value for VL-affected HH per 1,000 HHs/VL incidence per 10,000 persons in the control area. The effect is negative or positive if the VL-affected HH per 1,000 HHs/VL incidence per 10,000 persons is decreased or increased after intervention. Then the percentage reduction by intervention is calculated as (EI/[A]) × 100. ‡p values were calculated by Z statistic for pre- or post-rate differences between intervention and control areas. §Incidence per 1,000 HH. ¶Incidence per 10,000 persons.

The total number of household heads was 5,655, with 2,512 and 3,143 in the intervention and control areas, respectively. Before intervention, VL-affected households were 15.9 and 6.7 per 1,000 households in the intervention and control areas, respectively. After intervention, VL-affected households declined 13 times and 2 times, respectively, in the intervention and control areas compared with VL-affected households before intervention. The effect of the intervention in reducing VL-affected households in the intervention area compared with the control area was 70.5% by difference-in-difference analysis (Table 1). Again, using odds ratios in the longitudinal logistic regression model, we estimated the crude protection of households in the intervention area from VL by the intervention as 87% compared with those in the control areas. The protective effect of the intervention remained independent when adjusted for possible confounders (Table 2).

**Table 2:** Estimation of protection of households by the VL intervention using longitudinal logistic regression model with and without adjustment for confounders, Bangaladesh, 2006–2010*

Model; parameter	Odds ratio (95% CI)	Estimated protection by intervention at household level, % (95% CI)	p value
Simple, without adjustment for confounders; intervention	0.13 (0.030–0.557)	87 (44.3–97.0)	0.006
Full model, with adjustments for confounders			
Intervention	0.13 (0.03–0.56)	87 (44.3–97.0)	0.006
Family size >5 persons	1.75 (0.99–3.11)		0.054
HH head occupation, labor	2.38 (1.37–4.12)		0.002
Precarious (mud/thatched) house	4.64 (0.56–38.69)		0.156
HH head without any knowledge of VL symptom	0.25 (0.13–0.46)		<0.001
HH head without any knowledge of VL transmission	0.57 (0.33–0.98)		0.042
Having bed-net at home	0.49 (0.12–1.98)		0.319
Use of bed-net for protection against mosquito bites	2.57 (0.81–8.21)		0.109

*The intervention effect and covariates are tested in 2 different panel logistic regression models; simple not controlling for any covariates, full model controlling for confounders. VL, visceral leishmaniasis; HH, household.

## Conclusions

The community-based bed-net impregnation with slow-release insecticide significantly reduced VL incidence in VL-endemic areas. We used the difference-in-difference method for impact calculations because it is recommended by impact evaluation experts when effects of disease significantly differ between intervention and control, such as in our study (*5*–*10*). The protective effect was consistent and independent, as shown by the longitudinal logistic regression model. The differences in calculated effect and estimated protection at the household and community levels were due to households with multiple VL cases. Our findings agree with those of Ritmeijer et al. (*11*), who found a 59% reduction in VL by bed-net impregnation in Sudan. Our findings, however, were not consistent with those of Picardo et al. (*12*), who found no additional protection by random villagewise distribution of commercial insecticide–treated bed-nets compared with existing vector-control practice in India and Nepal. This discrepancy might be explained by the different delivery (commercial bed-net vs. existing bed-net impregnation) and coverage achieved (patchy villagewise vs. all villages in the area) by the intervention. We recommend mass coverage of VL-endemic villages with bed-net impregnation with slow-release insecticide for controlling VL in Bangladesh.

Technical Appendix Methods used to evaluate the effect of a community-based intervention with impregnation of existing bed-nets in reducing visceral leishmaniasis incidence in visceral leishmaniasis–endemic village of subdistrict Godagari, district Rajshahi, Bangladesh.
