# Supplementary material for: Reducing Visceral Leishmaniasis by Insecticide Impregnation of Bed-Nets, Bangladesh
Source: Emerg Infect Dis. 2013 Jul;19(7):1131–4. doi: 10.3201/eid1907.120932 (PMC3713966; doi:10.3201/eid1907.120932)
Supplement: Technical Appendix — Methods used to evaluate the effect of a community-based intervention with impregnation of existing bed-nets in reducing visceral leishmaniasis incidence in visceral leishmaniasis–endemic village of subdistrict Godagari, district Rajshahi, Bangladesh. [file 12-0932-Techapp-s1.pdf]

Article DOI: http://dx.doi.org/10.3201/eid1907.120932

Reducing Visceral Leishmaniasis by Insecticide Impregnation of Bed-Nets, Bangladesh

Technical Appendix

Methods Used to Evaluate the Impact of a Community-based Intervention with Impregnation of Existing Bed-Nets in Reducing Visceral Leishmaniasis Incidence in Visceral Leismaniasis–Endemic Village of Subdistrict Godagari, District Rajshahi, Bangladesh

Study Design, Study Area, and Population

The study design was a quasi-experiment with an intervention community and a control community. Visceral leishmaniasis (VL) incidence was measured before and after intervention from both intervention and control areas by repeated cross-section surveys.

The study was carried out in the Godagari upazila (subdistrict) of Rajshahi district, Bangladesh. According to the 2001 Census of Bangladesh, Godagarai has a total population of 279,545 living in 9 unions and 398 villages. A union is the smallest administrative unit in Bangladesh, and all public health–related activities are centered on the union basis. VL has been reported from only 5 (Deopara, Rishikul, Gogram, Pakri and Mohonpur) of 9 unions of the subdistrict. The total number of villages affected by VL in these 5 unions was 72 (out of 286): 36 in Deopara, 15 in Rishikul, 12 in Gogram, 6 in Pakuria, and 3 in Mohanpur. In Bangladesh, there have been no vector control activities since 1985 except in the malaria-endemic southeastern hilly part of the country, and there is no overlap of VL- and malaria-endemic areas. Since September 2011, the national program has introduced indoor residual spraying with Deltamethrin in VL-endemic villages.

Baseline Survey

From September 2006 through March 2007, trained field research assistants (FRAs) conducted a screening questionnaire by house-to-house visit in the 72 villages of the

abovementioned unions to detect patients in whom VL had been diagnosed over the preceding 12 months and those who had chronic fever (>2 weeks). In those fever cases, the trained FRAs examined for enlarged spleen, and in the positive cases, they performed the rK39 rapid test with Kala-azar Detect (InBios, Seattle, WA, USA). All persons positive for rK39 were referred to the subdistrict, district, or medical college hospital with a case referral form for further confirmation of splenomegaly and VL diagnosis. A case of VL was defined in accordance with the national kala-azar elimination program fever for >2 weeks, enlarged spleen, and rK39 rapid test positivity in a person from a VL-endemic area. The FRAs also conducted in-depth interviews with household heads by using a structured questionnaire in every 11th household and in households where they found past and present VL cases.

After the baseline survey, all 8,287 households (31,442 persons) in the Deopara union were invited to participate in the bed-net impregnation program. This was to simulate an eventual implementation of a VL vector control program by the national program in the union. There were 2,512 households from Deopara (11,426 persons) that were surveyed for VL incidence at baseline that constituted the study intervention area and a total of 3,143 households (14,021 persons) from VL-endemic villages in the other 4 unions without intervention that constituted the study control area.

The Intervention

During February–March 2008 a bed-net impregnation program with KO-Tab 1-2-3 was implemented in the Deopara union according to standard operational procedures, provided by the manufacture (Bayer Environmental Science, Bayer [Ply] Ltd., reg. no. 1968/011192/07, Isando, South Africa, CODE 05682036 C). Details about the dipping program can be found elsewhere (*1*). Briefly, the research team numbered all the households, collected information including the numbers of existing bed-nets in every household, and prepared a log against which number of dipped bed-nets was checked. The research team also trained public health personnel of the health system in bed-net dipping procedures, safety, and precautions. In each village, a village committee named “Kala-azar Nirmul Committee (village committee for VL elimination)” was formed. The committee selected the volunteers and bed-net dipping points. The public health personnel trained village volunteers how to educate villagers about bed-net dipping and how to conduct the dipping of bed-nets. Public health personnel informed villagers by house-to-house visits about the need for washing the nets before bringing them for dipping, about the procedures

of dipping, safety measures, and subsequent drying of the dipped nets in a horizontal position in a shaded area.

Follow-up Survey after 18 Months

In December 2009 and January 2010, a follow-up survey for active VL cases and past VL cases in the previous 12 months was conducted by using the procedures described above in all 72 villages.

Estimation of VL Incidence

Incidence per 10,000 was calculated by the number of VL cases (newly found during the survey plus those reported in the survey for the preceding 12 months) divided by the total population in the surveyed households multiplied by 10,000.

VL incidence at household level is expressed as number of VL-affected households per 1,000 households.

Sample Size Calculation

Sample size was calculated by assuming VL incidence per 10,000 people was 0.27%, expecting a 50% reduction of VL in the intervention area after intervention; setting the power of the study and the confidence limit of the estimation, respectively, at 80% and 95%. The required number of persons to be screened for active VL cases was 9,493 persons in each study area with a total sample size of 18,986. However, we surveyed a total population of 25,447 at baseline and follow-up, which gave sufficient power to our study.

Data Management and Statistical Analysis

A data entry program was developed by using Epi Info version 3.2.2 software (Centers for Disease Control and Prevention, Atlanta, GA, USA). Data were cleaned and checked for duplicates. Descriptive statistics were applied. Bivariate association was analyzed by using Pearson χ^2 or Fisher exact test where applicable. Z test was used to compare the estimated proportion between the intervention and control arm. Because the baseline statistics for outcome measurement differed significantly between intervention and control arm, we adapted a regression model to compare the rate of VL incidence and VL affected household. Comparative analyses were made at the population level as well as at the household level.

Effect of intervention (EI) was assessed by percentage reduction of VL incidence per 10,000 persons and VL-affected household per 1,000 households.

The EI was calculated on the basis of difference in differences analysis by using the following formula:

Effect of intervention (EI): (B-A) – (D-C)

where A = baseline value for VL incidence per 10,000 people/VL-affected households per 1,000 households in the intervention group; B = postintervention value for VL incidence per 10,000 people/VL-affected households per 1,000 households in the intervention group; C = baseline value for VL incidence per 10,000 people/VL-affected households per 1,000 households in the control group; D = postintervention value for VL incidence per 10,000 people/VL-affected households per 1,000 households in the control group.

The EI was negative or positive if the VL incidence per 10,000 people/VL-affected households per 1,000 households was decreased/increased after intervention and the effect was 0 if the VL incidence per 10,000 people/VL-affected households per 1,000 households was the same as at baseline. The percentage reduction of VL incidence per 10,000 people/VL-affected households per 1,000 households attributable to the intervention was calculated as (EI/[A]) × 100 and p value was calculated by using *Z* statistic as follows:

Z = D/SE where D = RD2-RD1 (RD1 and RD2 pre- and post-rate difference, respectively, for control and intervention areas); standard error, SE = √S (S = no. of event (VL cases or VL-affected HHs)/square unit (number of population/10,000) or (number of households/1,000) for each of 4 categories}.

Simple (Unadjusted) Model at Population and Household Level

The main outcome variables were “VL case” and “VL-affected households” before and 18 months after intervention. The outcome variable categorized as binary response (1 for VL case/VL-affected household and 0 for person without VL/household, not affected by VL). Based on the nature of the outcome variable, the longitudinal logistic regression model was used at population as well as at household levels to see whether the intervention significantly reduced the number of VL cases and VL-affected households, respectively. In the model, an interaction

term of being in the intervention arm at follow-up was included to estimate the effect of the intervention. The basic structure of the difference in differences regression model was:

Outcome = Intercept + a*Bed-net impregnation + b $\times$ Time + c $\times$ Interaction + error ..... (i)

where bed-net impregnation is 1 if it is the intervention area and 0 if it is the control area; Time is 1 if follow up and 0 if baseline; and interaction is 1 for intervention group at follow up.

Full (Adjusted) Model at Household Level

Within the sample of 5,655 households, a representative subsample of 556 households was used to collect the household socioeconomic and VL awareness data by using systematic random sampling. Variables with $p<0.20$ in the bivariate analysis on the subsample were considered as possible confounders and were extended to the 5,655 households database to develop the full model. It was found that the bionomial distribution fitted the data on subsample for the confounding variables. Therefore, extended sample for only confounding variables were made through the Bernoulli trail with only 2 possible random outcomes by using the probability (proportion) parameter estimated from the subsample (Technical Appendix Table). The outcomes were mutually exclusive and exhaustive. Then the extended sample was merged to the 5,655 household's database to develop a full model by using the same model structure (i) including the confounders for adjustment. The following variables were adjusted to determine how the intervention affected the household level: family size, household head occupation, housing condition (precarious house), household head knowledge on VL symptoms and VL transmission, having bed-net and use of bed-net.

In the table, odds ratio (OR) (95% CI) and its p value are given. Protection from VL disease were estimated as $(1 - OR) \times 100$ if $OR < 1$. Significances are stated at 5% level, and 95% CIs are given. For the data analysis, we used Stata 10.1 (Stata Corp LP, College Station, TX USA).

Reference

1. World Bank. Poverty reduction and equity. Estimation methods [cited 2011 Aug 28]. http://web.worldbank.org/wbsite/external/topics/extpoverty/extispma/0,,contentmdk:20188244~menupk:412148~pagepk:148956~pipk:216618~thesitepk:384329~iscurl:y,00.html

Technical Appendix Table. Sociodemographic characteristics and knowledge and practice about VL of HH heads in intervention and control areas, Bangladesh, 2006–2010

Indicator	Observed results, N = 556				Results after extension, N = 2,512			
	Intervention area, n = 254	Control area, n = 302	Total, N = 556	p value	Intervention area, n = 2,512	Control area, n = 3,143	Total, N = 5,655	p value
Mean age, y (SD)	42.1 (12.6)	43.3 (12.4)	42.8 (12.5)	0.22	42.7 (13.3)	42.7 (13.0)	42.7 (13.1)	0.95
Male respondents	242 (95.3)	290 (96.0)	532 (95.7)	0.66	2,337 (93.0)	2,934 (93.4)	5,271 (93.2)	0.63
Family size ≤5 persons	374 (68.3)	199 (65.9)	374 (67.3)	0.45	1,848 (73.6)	2,375 (75.6)	4,223 (74.7)	0.09
HH head without any education	134 (52.8)	155 (51.3)	289 (52.0)	0.74	1,318 (52.5)	1,609 (51.2)	2,927 (51.8)	0.34
HH head occupation, labor	73 (28.7)	108 (35.8)	181 (32.6)	0.08	719 (28.6)	1,146 (36.5)	1,865 (33.0)	**<0.0001**
HH head without any knowledge about VL symptoms	124 (48.8)	175 (57.9)	299 (53.8)	0.03	1,207 (48.0)	1,852 (58.9)	3,059 (54.1)	**<0.0001**
HH head without any knowledge about VL transmission	160 (63.0)	228 (75.5)	388 (69.8)	0.001	1,567 (62.4)	2,383 (75.8)	3,950 (69.8)	**<0.0001**
Have bed-net at home	247 (97.2)	296 (98.0)	543 (97.7)	0.55	2,439 (97.1)	3,072 (97.7)	5,511 (97.5)	0.13
Use of bed-net to protect against mosquitoes	227 (89.4)	274 (90.7)	501 (90.1)	0.59	2,254 (89.7)	2,866 (91.2)	5,120 (90.5)	0.063
Precarious house	231 (90.9)	299 (99.0)	530 (95.3)	**<0.0001**	2,280 (90.8)	3,115 (99.1)	5,395 (95.4)	**<0.0001**

*Values are no. (%) except as indicated. **Boldface** indicates significance. VVL, visceral leishmaniasis; HH, households.
